# At the heart of microbial conversations: endocannabinoids and the microbiome in cardiometabolic risk

**DOI:** 10.1080/19490976.2021.1911572

**Published:** 2021-04-25

**Authors:** Ramsha Nabihah Khan, Kristal Maner-Smith, Joshua A. Owens, Maria Estefania Barbian, Rheinallt M. Jones, Crystal R. Naudin

**Affiliations:** a Division of Gastroenterology and Hepatology, Department of Pediatrics, Children’s Healthcare of Atlanta and Emory University, Atlanta, Georgia, USA; b Emory Integrated Metabolomics and Lipidomics Core, Emory University, Atlanta, Georgia, USA; c Division of Neonatology, Department of Pediatrics, Children’s Healthcare of Atlanta and Emory University, Atlanta, Georgia, USA

**Keywords:** Endocannabinoid, microbiome, metabolic syndrome, cardiovascular, probiotic, sexual dimorphism

## Abstract

Cardiometabolic syndrome encompasses intertwined risk factors such as hypertension, dyslipidemia, elevated triglycerides, abdominal obesity, and other maladaptive metabolic and inflammatory aberrations. As the molecular mechanisms linking cardiovascular disease and metabolic disorders are investigated, endocannabinoids have emerged as molecules of interest. The endocannabinoid system (ECS) of biologically active lipids has been implicated in several conditions, including chronic liver disease, osteoporosis, and more recently in cardiovascular diseases. The gut microbiome is a major regulator of inflammatory and metabolic signaling in the host, and if disrupted, has the potential to drive metabolic and cardiovascular diseases. Extensive studies have unraveled the impact of the gut microbiome on host physiology, with recent reports showing that gut microbes exquisitely control the ECS, with significant influences on host metabolic and cardiac health. In this review, we outline how modulation of the gut microbiome affects host metabolism and cardiovascular health via the ECS, and how these findings could be exploited as novel therapeutic targets for various metabolic and cardiac diseases.

## The endocannabinoid system

For many, the use of plants and herbal remedies serve as their primary pharmacies. As was meticulously recorded in the world’s oldest known pharmacopoeia, the Pen-ts’ao Ching (The Herbal), *Cannabis sativa* (*C. sativa*) was used to alleviate an array of conditions, including intestinal constipation and rheumatism. Thus, cannabinoids constitute some of the oldest known medicinal compounds, with records of their earliest usage over 5,000 years ago in China. Cannabinoids are bioactive compounds isolated from *C. sativa*, known to produce a euphoric effect, and can also act as an analgesic, bronchodilator, appetite stimulant and muscle relaxant^1^. Since cannabinoids are primarily synthesized within plants, the term “endocannabinoid” was established for the endogenous molecules within mammals that bind and activate the same G-protein coupled receptors as cannabinoids. These receptors are known as the cannabinoid 1 (CB_1_) and cannabinoid 2 (CB_2_) receptors. This network comprising the CB_1_, and CB_2_ receptors, as well as the biologically active endogenous lipids that act on them, is known as the endocannabinoid system (ECS). The ECS has been implicated in several conditions, including chronic liver disease^2,3^ and osteoporosis.^4,5^ Additionally, in recent years, the term ‘endocannabinoidome (eCBome)’ has also been used to describe this complex signaling network. Particularly in the context of metabolic diseases, as the eCBome encompasses over 100 fatty-acid derived, endocannabinoid-related mediators and their subsequent target proteins and enzymes, many of which play critical roles in metabolic regulation and associated cardiometabolic complications like diabetes mellitus.^6,7^

CB_1_ and CB_2_ activation generate the production of downstream secondary messengers that can modulate the mitogen-activated protein kinases (MAPK), constituents of the nuclear factor NF-κB pathway, adenylate cyclase and specific ion channels.^8,9^ Thus, the ECS can have complex and diverse signaling effects. It is also important to note that endocannabinoids have been found to modulate other receptors besides CB_1_ and CB_2_, including intracellular receptors such as transient receptor potential (TRP) channels of vanilloid type-1, −2, −3, −4 or −8 (TRPV1-4, or −8),^8,10–13^ constituents of the transient receptor potential cation channel subfamily V, and the peroxisome proliferator-activated receptor -α and -γ (PPAR-α and PPAR-γ).^10^ Other CB receptor agonists, such as *N-*acylethanolamines (NAE), have also been found to activate other receptors, including G-protein-coupled receptor 55 (GPR55).^14,15^ In the adipose tissue of obese individuals, GPR55 expression is increased and this receptor continues to be studied in order to better understand its metabolic and cardiovascular effects.^16^ Notably, AEA can also inhibit T-type Ca^2+^ channels (Ca_v_3s), which play versatile roles in regulating various aspects of vascular smooth muscle function, such as pacemaker contractile activity.^17^ In addition, endocannabinoids can act intracellularly on mitochondrial CB_1_ receptors to regulate energy metabolism, with increased *in situ* endocannabinoids leading to decreased cellular respiration.^18^ Furthermore, CB_1_ and CB_2_ receptors differ in amino acid sequence and tertiary structure and have divergent tissue distribution. CB_1_ receptors are the predominant cannabinoid receptor found in the brain and its peripheral tissues, affecting motor activity, memory processing and sensory perception. CB_1_ receptors are also expressed in cardiac muscle, vascular smooth muscle cells, vascular endothelium, the kidney, liver hepatocytes, pancreatic β-cells and gastrointestinal tract.^19,20^ In contrast, CB_2_ receptors are mostly present on immune cells, and their expression is upregulated in response to the secretion of proinflammatory cytokines.^21,22^ CB_2_ receptors are also found in other peripheral organs, like the liver, where they have been found to play critical antifibrinogenic and immunoregulatory roles.^23,24^

Arachidonoylethanolamide (also known as anandamide; AEA) was among the first endogenous agonists of CB_1_ and CB_2_ receptors to be identified and has been found to induce many of the same pharmacological effects caused by Δ^9^-THC.^25^ While AEA is formed and released ‘on-demand’ by neurons in the brain, AEA can also be synthesized in the gastrointestinal tract as a degradation product of dietary arachidonic acid.^26^ *In vivo*, AEA can be generated from the membrane phospholipid, *N-*arachidonoyl phophotidylethanolamine (NAPE), via several reaction pathways mediated by *N-*acylphosphatidylethanolamine phospholipase D (NAPE-PLD). NAPE-PLD is also a critical for the synthesis of other endocannabinoids including 2-AG^27^ which exhibits similar CB1 and CB2 agonist activity to AEA.^28^

In addition to the ‘true’ endocannabinoids AEA and 2-AG, which are formed on-demand (as opposed to being stored in vesicles), other members of the *N*-acylethanolamines which have structural similarities to the ‘true’ endocannabinoids can affect the endocannabinoid response, although they do not directly bind to the CB_1_ and CB_2_ receptors. These endocannabinoid analogues include *N*-oleoylethanolamine (OEA, 18:1-EA), *N*-stearoylethanolamine (SEA, 18:0-EA), and *N*-palmitoylethanolamine (PEA, 16:0-EA), and are synthesized from the hydrolysis *N*-acylphosphatidylethanolamines.^29^ Saturated and monounsaturated *N*-acylethanolamines, like OEA, SEA and PEA do not bind to cannabinoid receptors; however, they play a critical physiological role and are known to activate the peroxisome proliferator-activated receptor α (PPAR-α), as well as other nuclear and extracellular receptors. By activating these receptors, saturated and monounsaturated *N-*acylethanolamines mediate a variety of effects, including appetite suppression, adipogenesis and inhibiting inflammation,^30^ and have also been implicated in the regulation of cancer cell proliferation.^31^

Through interactions with CB_1_ and CB_2_, AEA and 2-AG induce a myriad of bioactivities, known as the cannabinoid tetrad which includes hypothermia, catalepsy, hypo-locomotion and analgesia. Furthermore, activation of the cannabinoid receptors by AEA and 2-AG has been linked to a reduction in intraocular pressure and blood pressure, as well as bradycardia.^32,33^ Throughout various tissue types, 2-AG is detected at hundreds of times more abundant than AEA.^31^ Studies have shown that when fatty acid hydrolase (FAAH), an AEA-degrading enzyme, is inhibited or genetically deficient, the concentration of local AEA increases. This AEA abundance has a more potent influence in driving CB_1_-mediated activities than when AEA levels are low,^34^ suggesting divergent signaling effects based on the level of AEA present.

## The dual role of endocannabinoids and related molecules in inflammation

Many metabolic disorders, including those associated with cardiometabolic syndrome, are characterized by an increased systemic and cardiovascular inflammatory tone. The ECS is a complex endogenous signaling network and serves a critical role in regulating inflammation in a multitude of conditions, including celiac disease,^35,36^ irritable bowel syndrome (IBS)^37,38^ and colorectal cancer.^39–41^ Furthermore, the ECS has been upregulated in a number of inflammatory, neurodegenerative, metabolic and cardiovascular diseases, a phenomenon that has been suggested as autoprotective to hinder disease progression.^42^ Thus, the inhibition of endocannabinoid degradation to enhance endocannabinoid tone emerged as a potential treatment approach for various diseases. The two leading therapeutic approaches propose to inhibit the cellular uptake of endocannabinoids, or to inhibit the enzymes that catalyze endocannabinoid degradation, such as fatty acid hydrolase (FAAH) or monoacylglycerol lipase (MAGL).^25^

**Figure 1.**
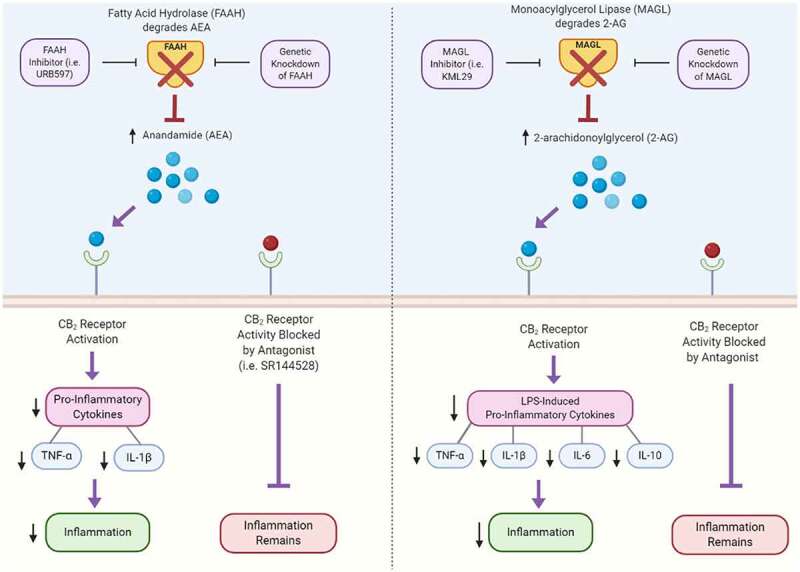
Anti-inflammatory signaling downstream of CB_2_ receptor activation

Endocannabinoids have been shown to help mitigate inflammation primarily through the activation of the CB_2_ receptor, which subsequently increases anti-inflammatory cytokines and decreases inflammatory cell levels in a variety of disease processes, including lipopolysaccharide (LPS)-induced pulmonary inflammation,^43^ trinitrobenzene sulfonic acid (TNBS)-induced colitis,^44^ experimental hepatitis^45^ and multiple sclerosis.^46^ Intraperitoneal injection of FAAH inhibitor URB597 or genetic knockout of FAAH resulted in decreased inflammation and a decrease in the inflammatory cytokines, tumor necrosis factor-alpha (TNF-α) and interleukin-1 beta (IL-1*β*).^47^ These anti-inflammatory effects were dependent on CB_2_ receptor activity since the responses were blocked with the addition of CB_2_ antagonist (SR144528).^47^When AEA levels were enhanced in *in vivo* models by a selective uptake inhibitor VDM-11, a reduced inflammatory tone in dinitrobenzene sulfonic acid (DNBS)-induced colitis was observed (Table 1).^58^ However, in this study, FAAH inhibition via AA-5-HT had no effect on either attenuating colitis or lowering inflammatory tone.^58^ Similarly, anti-inflammatory effects were observed when 2-AG levels were enhanced (Figure 1), including a decrease in LPS-induced levels of TNF‐*α*, IL-1*β*, IL-6, IL-10 expression in the frontal cortex, as well as a decrease in cell death in hepatic injury models (Table 1).^48,49^ Finally, inducing CB_2_ activity in mice reduced atherosclerotic lesions and decreased TNF‐ *α* and IL-6 levels (Table 1).^50^

**Table 1.** The dual roles of endocannabinoid signaling in inflammation

-	Model	Treatment	Receptor(s)	Effects	References
	LPS-induced inflammation & nociception	FAAH Inhibitor (URB597)	CB_2_	Decreased TNF-α & IL-1β levels Decreased inflammation	^47^
Anti-Inflammatory Effects	DNBS-induced colitis	AEA reuptake inhibitor (VDM-11)	CB_1_	Reduced inflammatory tone	^44^
	LPS-induced cytokine production in frontal cortex	MAGL Inhibitor (JZL184)	CB_1_, CB_2_	Decreased TNF-α, IL-1β, IL-6, & IL-10 levels	^48,49^
	*Apoe* -/- mice (atherosclerosis)	Low doses of THC which drives endocannabinoid-signaling via CB receptors	CB_2_	Decreased TNF-α & IL-6 levels Decreased atherosclerotic lesions	^50^
	Human leulkocytes	2-AG	CB_1_, CB_2_	Increased TNF-α secretion and leukocyte migration	^51–53^
Pro-Inflammatory Effects	CB_2_ -/- mice	Genetic knockout of CB_2_ in mice	Increased CB_1_ activity	Increased M1 (pro-inflammatory) macrophages in liver & adipose tissue	^54,55^
	FAAH -/- mice	FAAH inhibition & genetic knockout in mice	COX-2, LOX, & p450 oxidation	Increased pro-inflammatory prostaglandins	^56^
	Mast cells	2-AG	CB_2_	Increased TNF-α secretion	^57^

CB_2_ receptor activation by endocannabinoids, AEA and 2-AG, inhibit inflammation through reduced LPS and proinflammatory cytokines. While contradictory to the pro-inflammatory roles of endocannabinoids, in some settings it has been shown that inhibition of FAAH and MAGL (key enzymes that degrade endocannabinoids) can drive inflammation. These effects are CB_2_ receptor-dependent, as shown by the loss of anti-inflammatory effects through CB_2_ receptor blockade using CB_2_ antagonists (i.e. SR144528), whereas blockade of CB_1_ has no effect. Created with BioRender.com

**Figure 2.**
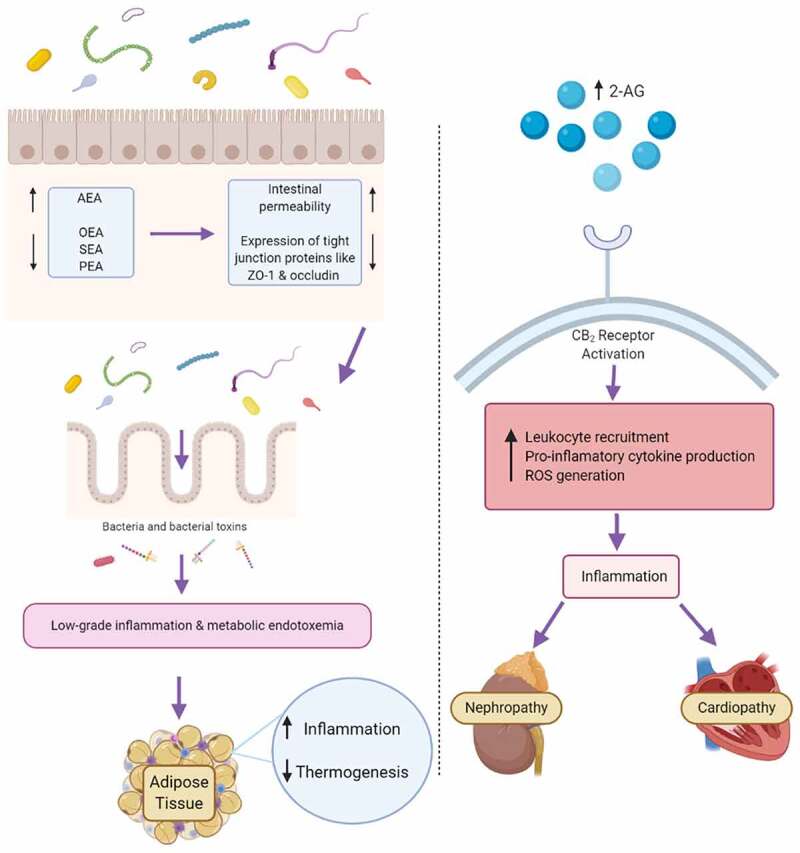
Pro-inflammatory signaling downstream of endocannabinoid receptor activation

Contrary to their role in responding to and inhibiting inflammation, cannabinoids have also been implicated as drivers of systemic inflammation. CB_1_ agonists like AEA have been referred to as ‘gate openers,’ as they induce gut permeability (Figure 2).^59^ The deletion of NAPE-PLD led to a decrease in *N*-acylethanolamines, like oleoylethanolamide (OEA), palmitoylethanolamide (PEA) and stearoylethanolamide (SEA) and was linked to increased inflammation in adipose tissue and is associated with increased macrophage infiltration and cytokine production, as well as a reduction in thermogenic browning activity.^60^ Interestingly, AEA levels were unaffected suggesting an alternative, compensatory synthesis pathway.^60^ Additionally, increased levels of 2-AG have been linked to an increase in the recruitment of leukocytes, pro-inflammatory cytokine secretion, and generation of reactive oxygen species (ROS).^51–54^ Many pro-inflammatory effects have been associated with increased 2-AG levels.^42^ For example, mast cells secreted more TNF‐ *α* when 2-AG levels were enhanced *in vitro* (Table 1).^57^ Increased endocannabinoid levels have also been found to drive ROS generation and enhance leukocyte recruitment in the onset of nephropathy and cardiomyopathy.^61,62^

Elevated endocannabinoid system tone can drive inflammation. Predominantly CB_1_ agonists like AEA can induce gut permeability, causing a ‘leaky’ gut. This can lead to the onset of metabolic endotoxemia as toxins such as LPS traverse the epithelial barrier to drive inflammatory signaling. 2-AG activation of CB_2_ receptors also has pro-inflammatory signaling effects in pathological settings, leading to leukocyte recruitment, pro-inflammatory cytokine production and ROS generation. Created with BioRender.com

It is also important to recognize that although AEA and 2-AG have been implicated in many disease states, they do not always exert their immunomodulatory effects through the cannabinoid receptors. For example, AEA and 2-AG can be metabolized to yield other lipid components, and thus serve as a source of arachidonic acid in the biosynthesis of other pro-inflammatory lipids.^42^ The inhibition of FAAH and MAGL increases the levels of endocannabinoids (since they are not being degraded) and leads to increased oxidation of endocannabinoids by cyclooxygenase-2 (COX-2), lipoxygenase (LOX) and p450 that drives inflammation.^42^ Consistent with this notion, increased pro-inflammatory prostaglandins were detected in FAAH knockout mice or conditions with reduced FAAH expression, suggesting increased oxidation of endocannabinoids by COX-2 (Table 1).^56^ Notably, LPS inhibits FAAH and promotes COX-2 expression,^63^ and these data, when taken together, suggests that endocannabinoids increase gut permeability in a positive, pro-inflammatory feedback loop.

Furthermore, when analyzing the dual roles of endocannabinoids in both driving and reducing inflammatory states, it is important to consider the redundancy in eCBome pathways.^64^ Not only can the same enzymes hydrolyze multiple different endocannabinoid substrates, but hydrolytic or oxidative metabolism of endocannabinoids can lead to the production of metabolites that are also capable of activating the same eCBome receptors.^64^ Thus, the role of eCBome redundancy in activating distinct receptors and altering metabolic enzyme expression may be particularly relevant when considering the conflicting inflammatory phenotypes associated with endocannabinoid signaling.

## Deducing the links between the endocannabinoid system, gut microbiome, and gut permeability

The ECS has been shown to mitigate inflammatory responses in gut diseases through host-bacterial interactions in *ex vivo* and *in vivo* models, with studies indicating a mechanism by which the gut microbiota can modulate the expression and activation of CB_1_ and CB_2_ receptors, as well as downstream inflammation.^65^ Increased permeability of the gut and intestine can be devastating, as leakage of Gram-negative bacterial components can lead to the onset of metabolic endotoxemia (i.e. a rise in plasma LPS levels), which can then exacerbate metabolic disorders like insulin resistance and diabetes.^66^ High-fat diets have been implicated in altering and impairing gut permeability and may catalyze the onset of systemic inflammation through the translocation of toll-like receptor (TLR) bacterial ligands to sub-epithelial compartments.^67^ The ECS has emerged as a potential avenue by which the gut microbiota modulates gut permeability, adipogenesis and plasma LPS levels.^67,68^ The ECS has been implicated in regulating intestinal permeability as endocannabinoids have been shown to enhance the expression of the tight-junction protein, occludin-1, while also decreasing the expression of claudin-1, another tight-junction membrane protein that serves as a paracellular barrier.^68,69^

Investigations into the mechanisms of how *Lactobacillus rhamnosus GG* (LGG) probiotics tighten the gut barrier demonstrate that these effects are dependent on ROS generation and subsequent activation of Nrf2 cytoprotective pathways.^70,71^ The effects of LGG may therefore require inhibition of ECS activity, since activation of CB1 or CB2 led to decreased expression or activity of the ROS producing enzyme, cyclooxygenase, and thus suppresses the production of intracellular ROS.^72^ It was also identified that increased intestinal expression of the satiety hormone leptin by LGG probiotic treatments is also ROS dependent,^73^ and thus may be inhibited by excessive ECS activition.

Notably, recent studies further implicate the role of gut microbiota in ECS signaling, as commensal microbe *Bacteroides* produces endocannabinoid-like *N-*acyl amides that structurally resemble OEA.^74,75^ These bacterial-derived endocannabinoid-like molecules also interact with receptors activated in the eCBome, specifically commendamide (N-acyl-3-hydroxypalmitoyl-glycine) was shown to activate G-protein-coupled receptors (GPCRs) G2A/GPR132.^74^

In genetically obese leptin-deficient mice (*ob/ob*) with metabolic endotoxemia arising from impaired gut barrier function, treatment with the CB_1_ antagonist SR121716A resulted in the partial rescue of the efficacy of the tight junction proteins, zonula occludens-1 (ZO-1) and occludin, as well as a decrease in plasma levels of LPS.^66^ Moreover, continual administration of the CB_1_ agonist, HU-210, over an extended period of four weeks led to an increase in bacteria-derived LPS and gut permeability in lean mice, as well as decreased mRNA expression of tight junction molecules, occludin and ZO-1.^68^ Co-treatment with the CB_1_ antagonist, rimonabant, negated the “gate-opening” effects of HU-210, whereas co-treatment with a CB_2_ receptor antagonist (SR144528) did not.^68^ Furthermore, inhibition of FAAH and MAGL and the addition of AEA and 2-AG increased gut permeability through a CB_1_-mediated mechanism.^76^ Thus, increased AEA levels are strongly linked to increased gut permeability. Additionally, emerging research has indicated that the administration of LPS led to a decrease in FAAH expression, while also enhancing AEA levels in human lymphocytes.^63,77^ This points to a pathway whereby LPS traversing a more permeable gut could potentially increase ECS tone and thus drive further leakiness of the gut epithelial barrier.

## The endocannabinoid system’s effects on gut motility

As the eCBome has been shown to have a multitude of effects in modulating energy balance and other metabolic complications, it is critical to consider the effects of endocannabinoids on gut motility. Oral THC administration has been previously shown to inhibit intestinal motility in a murine model^78^ and in humans.^79^ In mice fed a high-fat diet over a long-term period of eight weeks, exhibited increases in intestinal motility found to be associated with a rise in 2-AG levels and a decrease in AEA abundance.^80^ Increased AEA abundance and CB_1_ overexpression were observed in a mouse model of paralytic ileus (impaired gut motility induced by acetic acid), while a CB_1_ antagonist, SR141716A, mitigated this acetic-acid induced decrease in motility.^81^ Overall, most studies implicate AEA and CB1 activity in hypomotility, whereby providing further avenues for therapeutic exploration.

## The endocannabinoid system: a source of appetite stimulation

In addition to changing metabolic and inflammatory signaling in the body, the endocannabinoid system has also been linked to the regulation of appetite.^82^ Hypothalamic CB_1_ receptors continue to be studied for their role in the maintenance of energy balance, and their associations with the appetite-stimulating hormones, ghrelin and glucocorticoids, and the satiety hormone, leptin. Because endocannabinoids are generated *de novo* from the diet rather than stored in vesicles, their bioavailability also changes in response to restriction of food intake.^83^ When the endocannabinoids, AEA and 2-AG, were injected into the hypothalamus of rats, a consequent rise in food intake occurs through a CB_1_-mediated pathway.^84^ In humans, hedonic eating was associated with increased peripheral levels of both 2-AG and ghrelin, while AEA, PEA, and OEA levels decreased with time following the consumption of both pleasurable and non-pleasurable food.^85^ And more recently close links have been made between dietary lipid intake and endocannabinoid levels in both males and females.^86,87^ These results may account for the phenomenon commonly referred to as the ‘munchies,’ in which cannabinoid use is linked with an increase in food intake. Additionally, through interactions with the mesolimbic dopaminergic pathways, endocannabinoids promote the drive to seek high reward foods.^88^ When AEA was administered to the nucleus accumbens shell of rats, the CB_1_ receptor was activated, and an increase in the avidity and consumption of sucrose was observed. This behavior is similar to the hedonic response that is observed when Δ^9^-tetrahydrocannabinol increases dopamine release after sucrose exposure.^89^ In both *in vitro* and *in vivo* models, endocannabinoids activating the CB_1_ receptor were found to augment the neuronal response to sweet taste.^90^ Thus, because the CB_1_ receptor is highly expressed in regions of the brain associated with reward behavior and because endocannabinoid levels increase in the limbic system following instances of food deprivation, this data suggests that increased endocannabinoid tone drives the motivation to eat and enjoy foods.^84,91^

The cephalic phase of gastric secretion occurs before food enters the stomach, and ECS activity has been found to elicit a cephalic phase response that enhances anticipation for meals.^9,92^ In rats, upon high-fat liquid meal feeding, signals via the vagus nerve led to increased intestinal AEA and 2-AG.^93^ Moreover, this increased AEA and 2-AG was associated with increased food consumption, while a CB_1_ antagonist inhibited food intake.^93^ Furthermore, an enhancement in endocannabinoid tone is linked to an increase in the preferential uptake of palatable (high-calorie) food.^94^ In other studies, sham-feeding emulsions consisting of linoleic acid, oleic acid and unsaturated free fatty acids contributed to an increase in jejunum endocannabinoid levels.^95^ Furthermore, in a two-bottle sham-feeding preference test, rats showed a strong preference for the 18:2 free fatty acid option (high-reward diet), rather than the mineral oil, but this effect was not observed when the CB_1_ receptor antagonists, URB447 and AM6545, were administered.^95^ Thus, the ECS modulates energy balance through the regulation of feeding behavior and associated hormones in various neural circuits associated with reward-related behaviors, such as the hypothalamus.

To exert its appetite-stimulating effect, ghrelin requires efficient CB_1_ receptor signaling to activate AMP-activated protein kinase (AMPK),^96^ a critical enzyme that mediates the hormone’s function in the hypothalamus.^97^ Ghrelin failed to produce its appetite-stimulating effect in CB_1_-knockout mice, and also in mice treated with rimonabant (blockade of CB_1_).^96^ In wild-type mice, ghrelin activity led to an increase in the abundance of endocannabinoids in the hypothalamus.^96^ It is also important to note that both cannabinoids and ghrelin catalyze AMPK activity in the heart and hypothalamus, while also causing a reduction in AMPK activity in the liver and adipose tissue.^98^ AMPK is a heterotrimeric enzyme that helps to regulate energy balance and regulate the action of PPAR-γ, and is thought to mediate the action of metformin, a diabetes medication that inhibits gluconeogenesis in the liver. Furthermore, enhanced 2-AG and ghrelin levels have been correlated with an increase in food consumption in humans,^85^ while a rise in AEA abundance has been shown to elicit increased ghrelin production and secretion in rodent stomachs,^99^ suggesting an interplay between these two systems.

Glucocorticoids produced in the adrenal glands stimulate gluconeogenesis and thus play an important role in regulating insulin and glucose metabolism. Notably, glucocorticoids also stimulate endocannabinoid-mediated inhibition of the paraventricular nucleus (PVN) neuronal activity and thus inhibit the secretion of hormones from the hypothalamus.^100^ Fascinatingly, leptin (the “satiety” hormone) inhibits endocannabinoid-induced suppression of PVN activity.^101^ These effects may explain why enhancement of ECS tone in the hypothalamus was found to lead to peripheral insulin resistance, as well as a reduction in the efficacy of leptin.^100^ Furthermore, CB_1_ receptors have been shown to be necessary in glucocorticoid-driven metabolic syndrome, wherein CB_1_ knockout mice given chronic excess glucocorticoid exposure did not develop metabolic symptoms such as obesity, leptin dysregulation, hepatic steatosis and elevated blood triglycerides (unlike WT mice).^102^ Taken together, these data suggest an intricate balance between glucocorticoids and leptin, which is mediated by the ECS.

## The complex interplay between the endocannabinoid system and gut microbiome in cardiovascular disease

The ECS continues to emerge as an area of interest, particularly in regard to cardiovascular disease.^103,104^ The connection between cannabinoid use and cardiovascular disease was recently highlighted in a highly publicized statement from the American Heart Association.^105^ CB_1_ and CB_2_ receptors have been identified in human coronary endothelial and smooth muscle cells, and in the myocardium.^106,107^ The ECS serves a beneficial role in the cardiovascular system, as it decreases oxidative stress, neutrophil infiltration, and inflammation, while also stimulating other protective pathways through CB_1_ and CB_2_.^103^ CB_2_ deficiency appears to escalate atherosclerosis,^108^ and the CB_2_ receptor has been linked to the proliferation of vascular smooth muscle cells, decreases in oxidative stress and macrophage activation, as well as an overall enhancement of endothelial function.^109^ However, clinical administration of peripherally acting CB_1_/CB_2_ agonists led to adverse cardiovascular side effects.^110^ It is consistently found that inhibition of FAAH, the main degradation enzyme of AEA, drives both atherosclerosis and myocardial injury.^111–113^ The role of CB_1_ receptors in atherosclerosis and CVD is still being investigated and a source of much debate, as evidence of both pro- and anti-atherosclerotic activity exists.^107,114^

Inflammation is a particularly potent contributor to various cardiovascular disease etiologies, including coronary artery disease. Increased gut permeability and the subsequent onset of metabolic endotoxemia is caused by increased LPS levels in the plasma, and these conditions often give rise to the chronic low-grade inflammation found in atherosclerosis.^115–117^ Therefore, much attention has recently been devoted to the role of the gut microbiota potentially modulating the endocannabinoid system to catalyze the onset of atherosclerosis.^118,119^ Additionally, studies on rat heart models have shown that the administration of the CB_2_ receptor antagonist, SR144528, abrogated bacteria-derived LPS levels and heat-induced preconditioning against myocardial ischemia, while the CB_1_ receptor antagonist, rimonabant, failed to elicit the same effect.^119^ These results suggest that stress from heat shock or LPS may increase endocannabinoid production in inflammatory cells, which then can activate cardiac CB_2_ receptors downstream.

Furthermore, increased absorption of endotoxins across the intestinal barrier occurs in dysbiosis and in the disruption of mucosal barrier function. This can trigger CVD pathogenesis, since the injection of endotoxins has been reported to accelerate the onset of atherosclerosis in various animal models.^120^ As discussed previously, CB_1_ activation can disrupt barrier function.^68^ LPS leakage into systemic circulation leads to the downstream activation of TNF-α and IL-6 production, and increased circulating levels of pro-inflammatory cytokines. Thus, it is unsurprising that despite the known benefits of the ECS to cardiovascular health, there are several links between the gut microbiome, endocannabinoids and cardiovascular disease.^121–124^

**Figure 3.**
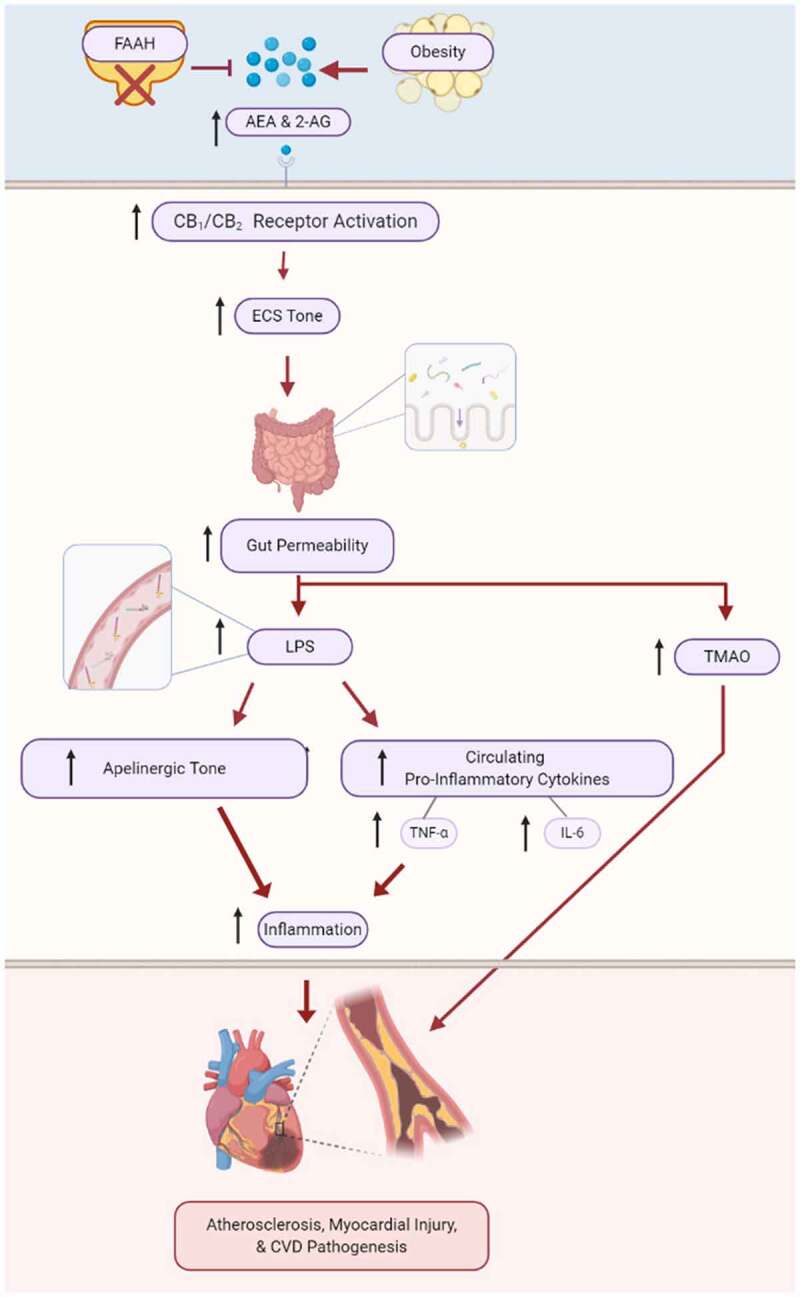
The role of endocannabinoids in cardiovascular disease pathogenesis

Changes in the gut microbiome and ECS in leptin-deficient mice (*db/db* mice), which have a mutation in the leptin receptor that confers susceptibility to obesity, insulin resistance and Type 2 diabetes, has been shown to affect apelin regulation in adipose tissue.^125^ Apelin is a recently discovered adipokine that has critical roles in maintaining cardiovascular health, including the regulation of heart contractibility, angiogenesis, blood pressure, cell proliferation and fluid homeostasis.^126,127^ Interestingly, obese mice also exhibit enhanced apelinergic system tone, in addition to enhanced ECS tone.^125^ The same increase in apelinergic system tone was observed when bacteria-derived LPS levels were also increased. While ECS tone appears to downregulate apelin and apelin G protein-coupled receptor (APJ) expression in lean mice, these effects are not observed in obese mice wherein LPS increases both apelin and ECS signaling. Adipose tissue treated with LPS and HU-210, a cannabinoid receptor agonist, saw an increase in apelin and APJ mRNA expression (Figure 3).^125^ Thus, these data help elucidate the regulatory role of inflammation and the complex crosstalk occurring between the gut microbiome and endocannabinoid system, and the importance of this conversation in the onset of associated cardiometabolic complications.

Endocannabinoid system tone is increasingly being linked to cardiovascular diseases. Aberrant or overactive endocannabinoid signaling, resulting from obesity or experimental inhibition of FAAH, has been shown to cause cardiovascular inflammation, atherosclerosis and myocardial injury. The major mechanisms proposed for these effects all result from increased gut permeability, causing elevated levels of circulating LPS and bacterial metabolite TMAO, which drives inflammatory signaling and increased apelinergic signaling. Created with BioRender.com

## Sexually dimorphic endocannabinoid signaling effects on the gut-cardiometabolic axis

Many studies of the sexually dimorphic effects of ECS signaling have focused on behavioral or emotional outcomes.^128^ For example, low-dose THC administration increased locomotor activity in female mice but not male mice,^129^ whereas higher-dose cannabinoid receptor agonism decreased locomotor activity specifically in females.^130^ However, very little is known about how metabolic and cardiovascular tissues are differentially affected by ECS activation in males versus females. It is not yet well-characterized how microbiome differences between males and females may impact these effects, and since metabolic and cardiovascular pathologies are also highly sexually dimorphic, further studies must be conducted to bridge this significant gap in the knowledge.

In studies of neurological tissues, CB_1_ receptor densities are generally higher in males in both rodent models and human subjects. This difference is equalized in mice after ovariectomy, suggesting that CB_1_ receptor density is controlled by estrogen expression.^131^ Similarly, the endocannabinoid 2-AG is expressed differentially in male and female neural tissues and shown to be regulated by the estrous cycle.^132^ Furthermore, endocannabinoids regulate hormone expression and secretion in females.^133^ Activation of CB_1_ and CB_2_ receptors in the hypothalamus leads to reduced secretion of reproductive hormones, including luteinizing hormone (LH)^134^ and gonadotropin releasing hormone (GnRH).^135^ This suppression of gonadal hormones by ECS activation results in reduced estrogen and progesterone release from the ovaries. Consistent with this notion, AEA activation of CB_1_ receptors expressed in the ovaries has also been shown to cause direct dysfunction of ovarian cells.^136^ Thus, there is bidirectional crosstalk occurring between the ECS and estrogen.

There are also differences in adipose distribution between males and females as endocannabinoids are lipophilic and may be sequestered in adipose tissues and released during lipolysis,^137^ likely contributing to the sex-specific effects of endocannabinoids on metabolism. CB_1_ activity in adipose tissue is also associated with ovarian dysfunction in polycystic ovary syndrome (PCOS).^138^ CB_1_ activity (but not CB_2_) is significantly elevated in adipose tissue of patients with PCOS.^138^ Notably, they also found that this increase correlated with insulin resistance.^138^ Insulin resistance and obesity are key drivers of PCOS, thus increased ECS signaling may represent a link between these conditions in female patients.

Additionally, the ECS has sexually dimorphic effects on gut motility and inflammation. THC administration in three individual dosages was sufficient to slow gastric emptying in women but not men.^139^ THC administration to Rhesus monkeys with simian immunodeficiency virus led to improved survival via inhibition of monocyte and macrophage-driven inflammation in the gut. However, no such effect was observed when female monkeys were tested.^140,141^ The mechanisms responsible for these effects remain largely unclear, and investigating sex-specific effects of ECS signaling on the gut-cardiometabolic axis is an important area for future study.

## The oral microbiome and salivary endocannabinoids: potential predictors of cardiometabolic complications

Changes in the oral microbiome and oral ECS tone have both been linked to the onset of cardiovascular disease.^142,143^ Saliva is considered the initial secretory fluid in the digestion process following the ingestion of food and has thus garnered interest as a site for the regulation of food intake and energy balance. The saliva consists of molecules derived from local and systemic sources and is composed of water primarily, as well as mucus, electrolytes, tasting enzymes, gastrointestinal hormones like ghrelin, and bacteria necessary for mucosal protection. Endocannabinoids including AEA and 2-AG, as well as other *N*-acylethanolamines like palmitoylethanolamide and oleoylethanolamide are also quantifiable in human saliva and are more abundant in obese patients.^144^ When analyzing salivary endocannabinoid and associated N-acylethanolamine levels in the fasting saliva and serum of normal-weight patients versus obese patients with insulin resistance, the obese/insulin-resistant patients had higher concentrations of circulating and salivary endocannabinoids.^144^ Furthermore, bodyweight reduction is also associated with a subsequent decrease in salivary AEA levels.^144^ Indeed, it was found that a 5% loss of bodyweight had an impact on salivary endocannabinoid levels, as a decrease in AEA levels was observed, but not in 2-AG and associated *N*-acylethanolamines [82]. The use of salivary endocannabinoids as surveillance for the onset of cardiometabolic diseases remains promising, as sample collection is simple and noninvasive. It remains to be determined whether salivary endocannabinoids are biomarkers that correlate not only with obesity but also with other cardio-metabolic phenotypes.

## Prebiotic and probiotic treatments for the therapeutic modulation of the endocannabinoid system

The modulation of the gut microbiome through prebiotics and probiotics is a viable avenue to control downstream ECS tone and which may prove important as novel therapeutic strategies for cardiometabolic diseases. Prebiotics and probiotics are commonly utilized as tools to alter the composition of the gut microbiome. Prebiotics are beneficial non-digestible food ingredients that stimulate microbiota activity, while probiotics are live bacterial cultures that are administered to benefit host health, including favorable effects across multiple organ systems. For example, *Lactococcus lactis subspecies cremoris* treatments in mice induce cytoprotective and anti-inflammatory activity in a gut colitis model^145^ as well as mitigate the deleterious effects of a high fat, high sugar diet on metabolism, in which a lowered expression of hepatic endocannabinoids was noted.^146^ In studies examining the role of the CB_2_ receptor in the gut, oral administration of *Lactobacillus acidophilus* (*L. acidophilus*) led to an upregulation of both μ-opioid and CB_2_ expression in the intestinal epithelium of rats, as well as a consequent decrease in visceral sensitivity.^147^ This effect was CB_2_-dependent since the blockade of CB_2_ receptors prevented this attenuation of visceral sensitivity.^147^ Thus, this study was the first to establish a microbial effect on host ECS tone.

The study of a mouse model with an intestine-specific knockout of myeloid differentiation primary response protein (MyD88), a key adaptor protein for Toll-like receptors, has offered further insight into how the ECS associates with intestinal dysbiosis and host metabolic health.^148^ It was found that mice deficient in MyD88 did not experience insulin resistance and the effects of diet-induced obesity, as they had lower fat mass, enhanced glucose metabolism and regulatory T cell proliferation, and a subsequent increase in energy expenditure.^148^ The observed beneficial effects also correlated with a reduction in AEA and 2-AG abundance.^148^ Significantly, the observed beneficial effects were transferrable by fecal microbiome transplantation and were therefore microbially mediated.^148^ Thus, it appears that enhanced ECS tone and the ensuing gut microbiome dysbiosis result in the divergent metabolic phenotype that causes the plethora of complications associated with many metabolic and cardiovascular disease etiologies.

Certain polyunsaturated fatty acids (PUFAs) have been shown to protect against inflammation and have been recommended as prebiotic supplements to aid in a variety of disease etiologies.^149,150^ Long-chain n-3 PUFAs (n-3 LCPUFAs) have been shown to improve hypertension, insulin resistance, liver steatosis, and heart steatosis. Diets rich in n-3 LCPUFA led to a reduction of fatty liver and inflammation in Zucker rats.^151^ These effects likely result from the observed decrease in the synthesis of arachidonic acid and its pro-inflammatory metabolites, and increased levels of docosahexaenoic acid and eicosapentaenoic acid.^152^ However, the exact mechanisms by which n-3 LCPUFA elicits these changes are yet to be elucidated. These results were consistent with another study where CB_1_ blockade with rimonabant led to reduced fatty liver and inflammation in Zucker rats.^153,154^ Thus, it could be speculated that dietary-introduced n-3 LCPUFAs may alleviate metabolic syndrome by reducing the bioavailability of endocannabinoid precursors, thereby also decreasing downstream ECS activation of CB_1_.

Blockade of the CB1 receptor (antagonist: SR141716A) in obese leptin-deficient (*ob/ob*) tightened the gut barrier and reduced plasma LPS, reduced weight gain, adiposity, blood glucose and hepatic inflammation.^68^ In the inverse experiment, it was shown that a chronic 4-week infusion of CB receptor agonist HU-210 using mini-pumps implanted subcutaneously into lean wild-type mice lead to increased plasma LPS.^68^ Altering the configuration of the gut microbiome through prebiotics, probiotics, and a high-fat diet all impact *Cnr1* (the gene encoding the CB_1_ receptor) expression in the colon.^68^ Furthermore, prebiotics modulate intestinal levels of endocannabinoids (via increased FAAH) leading to altered gut permeability and adipogenesis in obese leptin-deficient (*ob/ob*) mice.^68^

Outside of the gut, endocannabinoids directly regulate fat storage and their receptors, CB_1_ and CB_2_, are found on adipocytes and hepatocytes where they stimulate fatty acid synthesis. When comparing the adipose tissues of *ob/ob* mice to lean wild-type controls, a significant reduction in FAAH abundance and a significant increase in NAPE-PLD, the primary enzyme involved in AEA synthesis) as well as a corresponding increase in AEA levels, were observed.^68^ It was subsequently found that these changes were modifiable by the administration of prebiotics wherein colonic and adipose CB_1_ expression was reduced and FAAH expression was increased. Moreover, these prebiotic-induced changes correlated with decreased adiposity in *ob/ob* mice.^68^

Dietary supplementation of zebrafish with a probiotic mixture called VSL#3 (containing *Streptococcus thermophilus*, and various *Lactobacilli* and *Bifidobacteria*) increased ECS tone. These ECS effects include increased gene expression of endocannabinoid receptors *Cnr1, Cnr2* and *Trpv1*, increased gene expression of abhydrolase domain containing 4 (*Abhd4*) which is involved in AEA synthesis, and the decreased expression of FAAH and MAGL (enzymes involved in endocannabinoid degradation).^65^ This increase was associated with the induction of TLR signaling to regulate immune function.^65^ Another study demonstrated that irritable bowel syndrome patients supplementated with the probiotic, *L. acidophilus*, led to an increase in the expression of *Cnr2* mRNA (encoding the CB_2_ receptor) in colon epithelial cells.^147^ Additionally, another study found that *A. muciniphila* administration prevented fat mass gain, insulin resistance, adipose tissue inflammation and the onset of metabolic endotoxemia in mice.^155^ These beneficial metabolic effects were also associated with a consequent increase in the abundance of intestinal 2-AG.^155^ Moreover, *A. muciniphila* increased abundance of two eCBome lipids, palmitoyl-glycerol (1-PG) and 2-palmitoyl-glyerol (2-PG), with PPAR-α agonist activity.^156^ Interestingly, prebiotic supplementation of oligofructose in *ob/ob* mice led to decreased *Cnr1* mRNA expression along with decreased AEA synthesis, suggesting an overall reduction in ECS tone. This correlated with a consequent decrease in fat mass and an improvement in gut barrier function.^157^ Thus, probiotic and prebiotic supplementation may be a promising approach to regulate endocannabinoid concentrations and the balance of CB_1_ and CB_2_ receptors expressed in the gut.

## Conclusion and perspectives

As this review has demonstrated, the crosstalk occurring between the gut microbiome and the endocannabinoid system (ECS) appears to be bi-directional. The use of CB_1_ receptor antagonists has been explored as a means to treat diet-induced obesity and alleviate associated cardiometabolic complications.^82,158,159^ Furthermore, the administration of certain probiotics, such as *A. muciniphila*, has been shown to ameliorate visceral sensitivity and improve metabolic outcomes through enhanced CB_2_ expression.^155^ High-fat diets have also been shown to cause chronic low-grade inflammation and initiate a state of dysbiosis, and many obesity-related and cardiometabolic diseases appear to also be associated with both dysbiosis and enhanced peripheral CB_1_ signaling.^160,161^

Low abundant bioactive lipid species such as endocannabinoids have attracted attention as novel therapeutics. It is now known that endocannabinoids are key players in regulating inflammatory signaling in the host and that endocannabinoid levels influence outcomes in both cardiovascular and metabolic diseases. It was also recently discovered that endocannabinoids can be modulated by gut microbiota and by probiotic treatments. Since probiotics represent a safe and cost-effective therapeutic paradigm, it is critical to determine how to elicit modifications to ECS signaling through the administration of specific probiotics. Several bacterial species, as well as prebiotic or dietary shifts to the microbiome that modulate the endocannabinoid system, have already been identified. Further studies are needed to understand the specific microbial products and signaling effects that contribute to maintaining cardio-metabolic homeostasis and drive or prevent disease progression via the endocannabinoid system. The current knowledge of the crosstalk between the gut microbiota and the endocannabinoid system, and its subsequent impact on host cardiometabolic health has been demonstrated in this review article and others. Here, this review also explores these EC-microbiome interactions in the context of the role of salivary endocannabinoids in cardiometabolic diseases, as well as the sexually dimorphic effects associated with ECS signaling. Furthermore, we describe the relevance of these interactions both in cardiovascular and in metabolic diseases, since these are interlinked. Thus, we anticipate that future studies could be aimed at applying existing knowledge of these ECS and host-microbe interactions to develop novel therapeutic approaches to mitigate cardiometabolic risk.
